# Antibacterial Activity of *N*,*O*-Acylated Chitosan Derivative

**DOI:** 10.3390/polym13010107

**Published:** 2020-12-29

**Authors:** Agnieszka Piegat, Anna Żywicka, Agata Niemczyk, Agata Goszczyńska

**Affiliations:** 1 Department of Polymer and Biomaterials Science, Faculty of Chemical Technology and Engineering, West Pomeranian University of Technology, 45 Piastow Ave, 70-311 Szczecin, Poland; Agata.Goszczynska@zut.edu.pl; 2 Department of Microbiology and Biotechnology, Faculty of Biotechnology and Animal Husbandry, West Pomeranian University of Technology, 45 Piastow Ave, 70-311 Szczecin, Poland; Anna.Zywicka@zut.edu.pl; 3 Department of Materials Technology, Faculty of Mechanical Engineering and Mechatronics, West Pomeranian University of Technology, 19 Piastow Ave, 70-310 Szczecin, Poland; Agata.Niemczyk@zut.edu.pl

**Keywords:** hydrophobically modified chitosan, mucin, *Helicobacter pylori*

## Abstract

The antibacterial activity of *N*,*O*-acylated chitosan derivative with linoleic acid (CH_LA) was tested by disc and well diffusion, agar impregnation and microdilution methods against *Staphylococcus aureus*, *Escherichia coli* and *Helicobacter pylori* strains. Hydrophobically modified chitosan (HMC) was expected to exhibit enhanced antibacterial activity and specific mucin interactions. Although diffusion tests have not indicated the antibacterial potential of chitosan (CH) or CH_LA, the results of the microdilution method demonstrated that tested polymers significantly reduced the amount of living bacteria cells in different concentrations depending on the microorganism. Additionally, CH_LA was characterized by enhanced antibacterial activity compared to CH, which may suggest a different mechanism of interaction with *S. aureus* and *H. pylori*. Furthermore, the UV-VIS analysis revealed that the amphiphilic character of derivative led to strong CH_LA–mucin interactions. The study proved the high potential of CH_LA in antibacterial applications, especially for the gastrointestinal tract.

## 1. Introduction

Chitosan is a polysaccharide produced by deacetylation of chitin. This reaction leads to the characteristic copolymer structure of this biopolymer that consists of D-glucosamine (GlcNH_2_) and N-acetyl-D-glucosamine (GlcNHAc) units. The molar fraction of these units depends on the deacetylation reaction and is defined by the degree of deacetylation. On the one hand, the presence of amino groups from GlcNH_2_ units and their susceptibility to protonation are responsible for chitosan properties such as solubility in acidic aqueous media, and inter- and intramolecular electrostatic repulsions in solution, that is, for its hydrodynamic behavior. On the other hand, some properties, such as ions chelation or action with free radicals, are the consequence of the presence of both amino and hydroxyl groups (from GlcNH_2_ and GlcNHAc), in the chitosan main chain. This double functionality allows chemical modification of chitosan, resulting in different solubility of chitosan derivatives, [1] with distinct abilities to interact with different biologically active molecules through hydrophobic, hydrogen bonding or Van der Waals forces.

The increasing resistance of bacteria to antibiotics, together with the greater awareness of the side effects of long-term antibiotics therapy, is driving the research towards the development of alternative methods for bacteria eradication. In this sense, chitosan is one of the natural-based polymers with already proved antiviral [2,3], antifungal [4,5,6] and antimicrobial potential. Its broad application range as an antibacterial or bacteriostatic agent is a result of the direct interaction of chitosan macromolecules with bacterial cells as well as the ability of this biopolymer and its derivatives to produce various drug delivery systems [7,8]. The proposed mechanism of direct chitosan antibacterial action depends on the type of bacteria (Gram-positive, Gram-negative) and the physico-chemical properties of chitosan, e.g., molecular weight or degree of deacetylation. High molecular weight chitosan interacts with the outer cell wall of the bacteria, creating an impermeable coating preventing the nutrients and metabolic by-products exchange, and thereby causing cell death. This is the typical mechanism of interaction with Gram-positive bacteria characterized by thick cell walls. For Gram-negative bacteria possessing a much thinner cell wall, it is preferable to use low molecular weight chitosan. In this specific situation, short macromolecules can go across the cell membrane interacting with negatively charged cell elements, like DNA phosphate residues, and blocking the transcription reaction and mRNA synthesis [9]. Regarding chitosan derivatives, an enhanced antibacterial activity has been mainly reported for the quaternized chitosan, due to the increased positive charge density and stronger complexation with the cell membrane [10]. Water-soluble arginine-modified chitosan has been also stated to inhibit the growth of Gram-negative bacteria [11], although no direct lysis of *Escherichia coli* was observed. Bacteria contacted with chitosan-arginine macromolecules tended to aggregation and their cell envelopes were permeabilized. Also *N*-benzoyl-*O*-acetyl-chitosan [12], ethylene diamine tetraacetic acid grafted chitosan and carboxymethyl chitosan have demonstrated antimicrobial activity against negatively charged bacteria [13].

Depending on the potential application, chitosan can be modified to obtain water-soluble materials [14,15] or so-called hydrophobically modified chitosan (HMC), which is highly prone to self-assembly in aqueous solutions. Films from HMC obtained by solvent evaporation demonstrated antibacterial activity against *Escherichia coli*, *Bacillus subtilis* and *Staphylococcus aureus* [16], and HMC sponges successfully removed bacteria directly from the suspension [17]. HMC was also used to encapsulate hydrophobic anticancer drugs [18,19] or antibiotics used to treat bacterial wound infections [20].

The attractiveness of chitosan in antibacterial applications is also related with its mucoadhesive properties. Several studies have proved the strong interactions between positively charged chitosan macromolecules and negatively charged mucins presented in mouth, intestinal and genital tracks [21,22]. This semipermeable, hydrogel-like structure is the first barrier that protects the internal bodily surfaces against water loss and attack of different pathogens. In a view of that fact, the preservation of mucoadhesiveness of chitosan derivatives would be the added value in terms of antimicrobial action. Unfortunately, mucoadhesiveness of HMC is rarely studied and the interaction mechanism among mucins and chitosan is not well-known. Considering that HMCs were used as drug delivery systems with different proteins, e.g., polypeptidic insulin [23,24] or antiangiogenic small peptide drugs [25,26], the interaction with mucin-type glycoproteins are also expected, especially those exhibiting amphiphilic character (MUC2, MUC5B, MUC6).

Therefore, the aim of the presented work is to study the antibacterial activity as well as mucoadhesiveness of hydrophobically modified chitosan in comparison with unmodified chitosan. Additionally, diminished amount of amino groups in HMC on these properties has been discussed. The antibacterial activity was investigated against three bacteria strains: *S. aureus*, *E. coli* and *H. pylori* by diffusion and microdilution methods. The UV-VIS spectroscopy was used to determine the interactions of chitosan and HMC with mucin type II molecules evaluating the influence of hydrophobic chains on the mucoadhesiveness.

## 2. Materials and Methods

### 2.1. Materials

Chitosan (CH) ChitoClear^®^ 43000—hqg10 was purchased from Primex ehf Iceland Company(Siglufjordur, Iceland) (deacetylation degree ~83%, determined by the ^1^H NMR, Bruker, Billerica, Massachusetts, USA; weight average molecular weight: 145.7 kDA determined by GFC, gel filtration chromatography, Shimadzu, Japan, Shim-Pol, Warsaw, Poland). Linoleic acid (LA) (≥99%) 1-ethyl-3-(3-dimethylamino–propyl) carbodiimide hydrochloride (EDC), N-hydroxysuccinimide (NHS), hydrochloric acid conc. (HCl), formic acid conc. (FA), methanol (MeOH), sodium tripolyphosphate (TPP) and mucin type II from porcine stomach (MUC; bound sialic acid ≤1.2%) were purchased from Sigma Aldrich Co., Ltd (Poznan, Poland). and used without further purification. Dichloromethane (DCM, analytical grade) and ethylene glycol (EG, analytical grade) were purchased from Chemland, (Stargard, Poland).

### 2.2. Synthesis of CH_LA

Chitosan derivative was synthesized and characterized as described previously [27]. Briefly, reaction was conducted at pH = 4.0 (aqueous HCl solution), at room temperature. First, chitosan was dissolved in hydrochloric acid/methanol mixture, at the concentration of 1 wt/v%, under continuous stirring for 24 h. After this time, linoleic acid (LA) (30 wt%) was dissolved in 30 mL of methanol and an activation step with EDC was performed for 20 min. In the next stage, solution of activated LA was added dropwise to the chitosan solution and stirred for 24 h. In order to purify the product, the reaction mixture was dialyzed against water for 48 h at 25 °C (Spectra/Por^TM^ 3, MWCO: 3.5 kDa, Gardena, CA, USA) and then freeze-dried (freeze dryer, Christ, Alpha 1-2, Martin Christ, GmbH, Osterode am Harz, Germany ) for 48 h. The lyophilized CH_LA was in yellowish, solid, sponge-like form. A weight average molecular weight of 65.8 kDa was obtained by GFC [27].

### 2.3. Dynamic Light Scattering

The hydrodynamic diameter of chitosan and derivatives was evaluated at several pH by dynamic light scattering (Zetasizer NanoZS, Malvern Instruments Ltd., Malvern, UK). All measurements were carried out at 25 °C with the backscatter detection system at 173° angle. The excitation source was a helium–neon vertically polarized laser operating at a wavelength of 633 nm.

### 2.4. Polymer—Mucin Interactions

Mucin, CH and CH_LA were dissolved in 0.1 M HCl at a constant concentration of 1 mg/mL. All solutions were dissolved for 24 h at 37 °C. Mucin and CH were soluble in 0.1 M HCl, whereas CH_LA was partially soluble and formed an opaque solution/dispersion. Mixtures containing 1:1 and 1:2 polymer: mucin weight ratios were mixed in a quartz cuvette and gently stirred with magnetic stirred in UV-Vis spectrophotometer (Jasco, V-630, Tokyo, Japan) 5 min before measurements. The measurements were repeated after 24 h, while the analysis mixtures were incubated in a laboratory shaker (Heidolph Polymax 1040, Schwabach, Germany) at 37 °C between measurements. The turbidity of the mixture solutions was measured at 600 nm.

### 2.5. Bacteria Optimal Cultivation Conditions

The antimicrobial activity of CH and CH_LA was tested against *Staphylococcus aureus* DSM 1104, *Escherichia coli* DSM 1103 and *Helicobacter pylori* DSM 21031 (Deutsche Sammlung von Mikroorganismen und Zellkulturen—German Collection of Microorganisms and Cell Cultures). Prior to the experiment, the *S. aureus* and *E. coli* were plated onto the BHIA (Brain Heart Infusion agar, BioMaxima, Lublin, Poland) and cultivated overnight at 37 °C. After the incubation, one colony forming unit (CFU) of each microorganism was transferred into 10 mL of BHI and incubated overnight under the same culture conditions while shaking.

*H. pylori* was plated onto the Brucella Blood Agar (BBA, BioMaxima, Lublin, Poland) and incubated at 37 °C for 3 days under microaerobic conditions (6% O_2_, 7% CO_2_, 7% H_2_, 80% N_2_). After the incubation, one colony was transferred into 10 mL of Brucella Broth medium with 5% horse serum and incubated 48 h under the same culture conditions.

### 2.6. Antibacterial Activity of Chitosan and Chitosan Derivatives

#### 2.6.1. Disc Diffusion Method

Standard paper discs (6 mm, Biomaxima, Lublin, Poland) were impregnated with CH or CH_LA solutions of 10, 5, 2 and 1 mg/mL in phosphate buffered saline (PBS, Sigma-Aldrich, Poznan, Poland) and placed onto the surface of the Mueller-Hinton (M-H) agar medium (BioMaxima, Lublin, Poland) seeded with the suspension of *S. aureus* or *E. coli* at a concentration of 5 × 10^5^ CFU/mL. Additionally, a paper disc impregnated with sterile PBS was used as a negative control. The cultures were carried out at 37 °C for 24 h. In the case of *H. pylori,* bacteria were seeded on BBA and incubated 48 h under microaerobic conditions (6% O_2_, 7% CO_2_, 7% H_2_, 80% N_2_). After incubation the average diameter of the inhibition zone (in mm) was evaluated. The test was performed in triplicate.

#### 2.6.2. Well Diffusion Method

Well diffusion method was performed as a modification of the disc diffusion method. Instead of paper discs, wells (6 mm *diameter)* were cut out from agar using a cork borer and filed with 10 µL of the CH or CH_LA solutions in PBS (10, 5, 2 and 1 mg/mL). The plates with *S. aureus*, *E. coli* or *H. pylori* were incubated under optimal conditions as described above. After incubation the average diameters of the inhibition zone (in mm) were evaluated. The test was performed in triplicate.

#### 2.6.3. Agar Impregnation Method

Agar plates were impregnated with different concertation of CH or CH_LA solutions in PBS (10, 5, 2 and 1 mg/mL). Next, 100 µL of bacteria suspension (*S. aureus*, *E. coli* or *H. pylori*) at concentration of 5 × 10^5^ CFU/mL was seeded using plaques on agar plate. The plates were incubated under optimal conditions specified above. After incubation the CFU were calculated. Results were compared to the control—non impregnated agar plate.

### 2.7. Microdilution Method

Antibacterial properties of CH and CH_LA were assessed by microdilution method. At the first step the pH of bacteria culture medium (BHI for *E. coli* and *S. aureus* and BBA for *H. pylori*) was adjusted to 4, 4.5, 5, 5.5, 6 with 1 M HCl. CH and CH_LA was dispersed in PBS at concentration of 40 mg/mL (initial concentration). Subsequently, 100 μL of medium and 100 μL of CH or CH_LA were added to the first of the wells of 96-well titration micro-plates (Becton Dickinson and Co., Franklin Lakes, NJ, USA). After that a two-fold serial dilution of CH and CH_LA was performed up to the final concentration of 0.039 mg/mL. Next, 100 μL of the bacterial suspension (1 × 10^5^ CFU/mL) in the media at adjusted pH was introduced to each well. The entire plate was incubated under above mentioned optimal condition. After incubation (24 h for *S. aureus* and *E. coli*, 48 h for *H. pylori*, respectively) 100 µL of the bacterial suspension was transferred to a 96-well plate and simultaneously 10 µL of 3-(4,5-Dimethylthiazol-2-yl)-2,5-diphenyltetrazolium bromide (MTT) solution (3 mg/mL in PBS, Sigma-Aldrich, Poznań, Poland) was added into each well. The plate was incubated at 37 °C for 30 min. In the next step, 100 µL of isopropanol was added to each well and the plates were vigorously shaken for 15 min. The amount of MTT formazan formed during the incubation was measured at the wavelength of 570 nm, and with 690 nm as reference wavelength, using a microplate reader (Infinite 200 PRO NanoQuant, Tecan, Männedorf, Switzerland). The experiment was conducted in technical triplicates and repeated three times (three biological replicates). The results are shown as % of living cells in the presence of CH or CH_LA, compared to positive control (bacteria with medium) calculated following Equation (1):

(1) $$\%\;\mathrm{living}\;\mathrm{cells}=\;\frac{{\left({\mathrm{Abs}}_{S}-{\;\mathrm{Abs}}_{B}\right)}_{\;}\;}{({\mathrm{Abs}}_{C}-{\;\mathrm{Abs}}_{B)}}*100\%$$

where, Abs is the absorbance difference, i.e., the absorbance at 570 nm diminished by absorbance at 690 nm; S, medium with bacteria and CH or CH_LA; C, medium with bacteria (positive control); B, pure medium (blank).

### 2.8. Statistical Analysis

Data are shown as means ± standard errors of the means (SEM) obtained from at least three different measurements (plus technical repetitions). Statistical differences between different BC samples were determined by one-way analysis of variance (ANOVA) and Tukey’s post hoc test. All analyses were considered statistically significant when the P value was less than 0.05. The statistical analyses were conducted using Statistica 9.0 (StatSoft, Cracow, Poland).

## 3. Results

### 3.1. Synthesis and Chemical Structure Analysis

**Figure 1:**
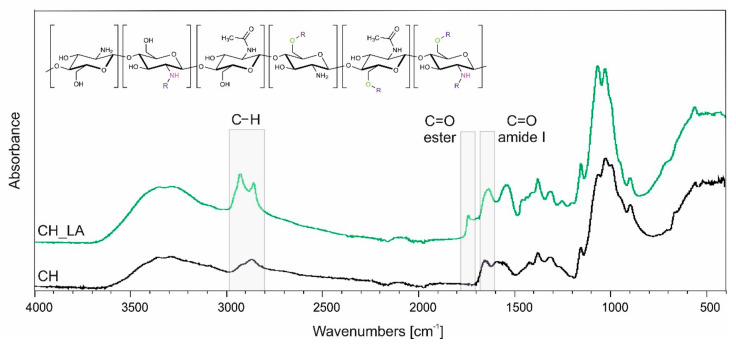
Infrared spectra of chitosan (CH) and chitosan with linoleic acid (CH_LA) and chemical structure.

Synthesis of chitosan derivative was successfully performed according to our earlier procedure [27]. Conducting the reaction between chitosan and linoleic acid in water adjusted to pH = 4.0 using HCl with equimolar amount of EDC as a coupling agent, leads to *N*,*O*-acylated chitosan derivative with altered solubility comparing to the unmodified chitosan. Figure 1 shows the FTIR spectra of CH and CH_LA with marked characteristic bands of *N*,*O*-acylated product and chemical structure of derivative.

The main differences between the spectra are observed for bands related to: alkyl stretching and bending in the 3000−2840 cm^−1^ region; amide I stretching mode at 1640 cm^−1^ and ester stretching mode around 1740 cm^−1^ being confirmation of *N-* and *O-*acylation, respectively. The other bands observed are: newly formed 1200 cm^−1^ and 1250 cm^−1^ bands characteristic for ester stretching mode of C=O and C−O; band at 1150 cm^−1^ related to asymmetric bridge oxygen stretching; band at 1425 cm^−1^ (CH)/1435 cm^−1^ (CH_LA) assigned to CH_2_ and CH_3_ vibration. Detailed structure description can be found in our earlier work [27].

### 3.2. Mucin Interactions

**Figure 2:**
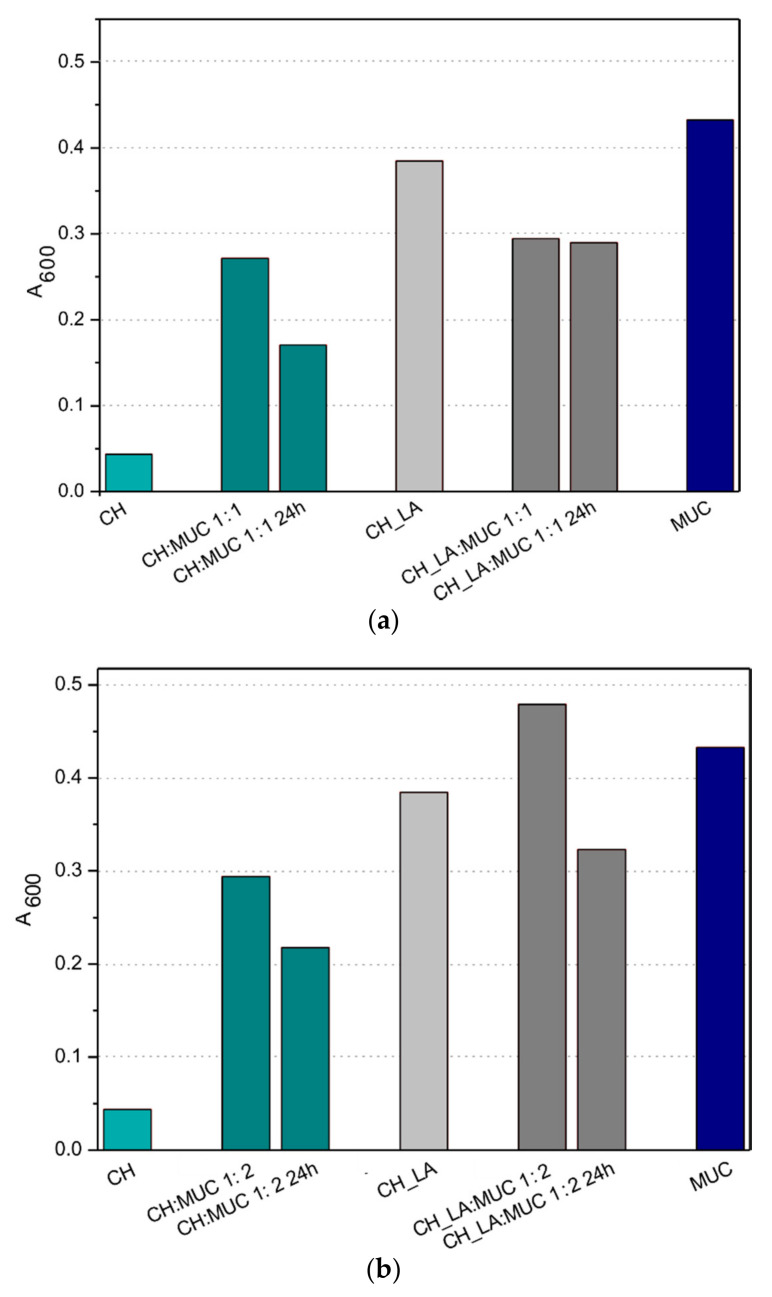
Dependence of absorbance of CH and CH_LA solutions in contact with mucin for systems with (**a**) 1:1 and (**b**) 1:2 weight ratio (CH:MUC).

The analysis of the mucoadhesive properties of CH and CH_LA was performed in contact with mucin type II. CH, CH-LA and mucin solutions were used as a reference. Figure 2 presents the results of measurements of absorbance at the wavelength of 600 nm for solutions of CH and CH_LA in contact with mucin, at 1:1 and 1:2 weight ratio. Two measurements were carried out, the first after 5 min from obtaining the mixtures and the second after 24 h of their incubation at room temperature. Among neat solutions the lowest absorbance was noticed for unmodified CH and the highest for mucin solution. In general, the changes in the absorbance could be attributed to formation of CH—mucin or CH_LA—mucin aggregates or even precipitation of the interaction products [28,29].

As presented in Figure 2 the absorbance of chitosan solutions have comparable values for systems with 1:1 (Figure 2a) and 1:2 (Figure 2b) weight ratio (CH:MUC) at both time points. This suggests that regardless of the weight ratio the charges of $NH_{3}^{+}$ of chitosan and $SO_{4}^{2-}$ of sialic acid units of mucin in 0.1 M HCl are compensated and the CH_MUC aggregates exhibit similar hydrodynamic behavior over time, which is also in agreement with the Rossi observation [28].

For chitosan derivative at 1:1 weight ratio, the absorbance (Figure 2a) is almost constant over time, which might suggest that the CH_LA_MUC complex, at selected experimental conditions, is hydrodynamically stable and reaches the equilibrium just after mixing, suggesting proportional hydrophobic interactions. The opposite behavior is observed for 1:2 CH_LA_MUC system (Figure 2b). In this case, at the beginning the absorbance is significantly higher than for the rest of solutions and subsequently, after 24 h, the absorbance decreased below the values characteristic for neat CH_LA or neat mucin. High absorbance for the CH_LA_MUC system suggests that at the beginning, the properties of both polymers mixed impose thermodynamic incompatibility. Nevertheless, after some time (24 h) the stability of the system (revealed as decreased absorbance) is achieved.

### 3.3. Antibacterial Activity of Chitosan

#### 3.3.1. Diffusion Based Methods

**Figure 3:**
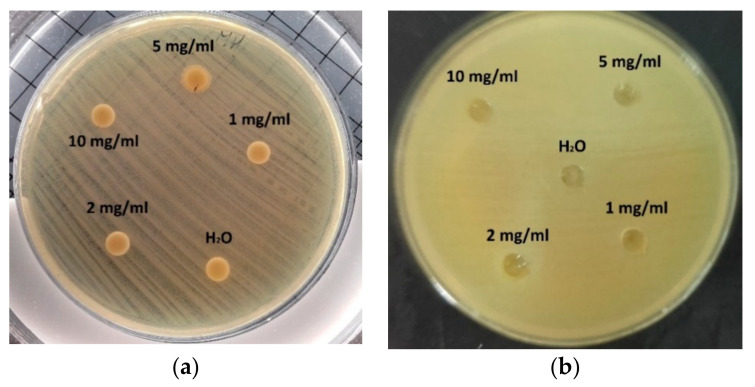
Antimicrobial activity of CH against *S. aureus*: (**a**) disc diffusion method, (**b**) well diffusion method.

For disc diffusion and well diffusion methods no inhibition zones were observed at any concentration of CH nor CH_LA and neither for any tested bacteria (Figure 3). This fact can be attributed to the limited diffusion ability of chitosan macromolecules through the agar hydrogel structure. Chitosan molecules acts on microorganism only in direct contact. The diffusion is even more restricted for CH_LA macromolecules due to the hydrophobic modification. Presented in literature positive results for antimicrobial activity of chitosan employing these methods have been mainly reported for chitosan with lower molecular weight or water-solubility properties as well as for chitosan films in direct contact with the agar surface [30,31,32,33].

Similarly, the agar impregnation test did not provide the proper confirmation of the antibacterial activity of either CH or CH_LA for any tested concentration (Table 1). There were no statistically significant differences between control and tested materials.

For the above discussed methods, diffusion to solid agar was the limiting process preventing the proper antibacterial properties evaluation. Therefore, in view of the fact that the polymer should be directly contacted with the bacteria (to do not restrict the diffusion process), the microdilution method was applied, where the CH or CH_LA is suspended in the liquid media with bacteria. In such test the diffusion process in not the limiting factor influencing the polymer-bacteria interactions.

#### 3.3.2. Microdilution Method

To evaluate the antibacterial properties of CH and CH_LA the microdilution method at various pH levels was conducted. It is known that chitosan antibacterial activity depends on the pH of the environment [34] since it influences its hydrodynamic state (higher/lower solubilization) and protonation degree. Additionally, bacteria strains also exhibit pH sensitivity, therefore in the first step the influence of pH on used bacteria strains was determined. The optimal pH for all tested microorganisms is in the range of 5–7, as presented in Table 2. The obtained results are in agreement with literature data concerning *E. coli* and *S. aureus* cultivation [35]. For *H. pylori* it is indicated that the optimum pH is 7 and at pH lower than 4.5 bacteria become irreversibly inactivated [36].

**Figure 4:**
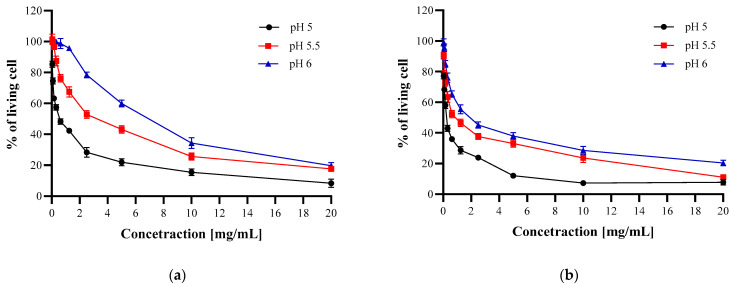
Antibacterial properties of CH (**a**) and CH_LA (**b**) against *S. aureus* at different pH.

**Figure 5:**
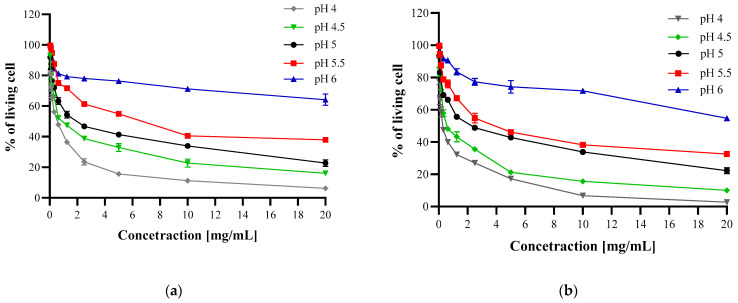
Antibacterial properties of CH (**a**) or CH_LA (**b**) against *E. coli* at different pH levels.

**Figure 6:**
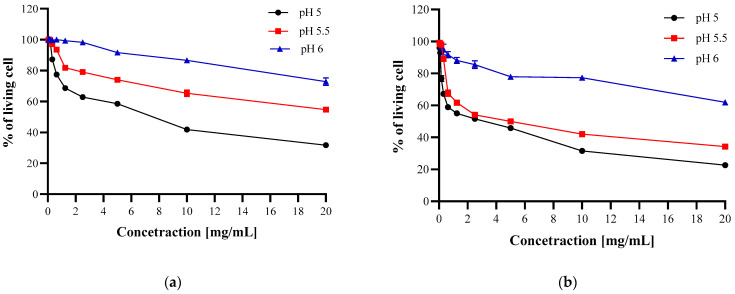
Antibacterial properties of CH (**a**) or CH_LA (**b**) against *H. pyroli* at different pH.

The results (Figure 4, Figure 5 and Figure 6) confirmed that CH and CH_LA possess antibacterial properties and are active against Gram-positive (*S. aureus*) and Gram-negative (*E. coli*, *H. pylori*) bacteria. In general, the antimicrobial effect increases with higher CH and CH_LA concentration and lower media pH, which stays in the line with the findings of other authors [34,35,37]. For example, at pH 5 and the highest concentration the average % of living bacteria cells is 8% for *S. aureus*, 22% for *E. coli* and 32% for *H. pylori* (Figure 4, Figure 5 and Figure 6, Tables S1–S3, Supplementary Materials).

As can be observed in Figure 4 at the highest concentration (20 mg/mL) the percentage of living *S. aureus* cells is on a similar level for both materials at all pH values (Table S1). Although the final antibacterial effect of both materials is comparable, it can be expected that the interaction with bacteria will be based on different mechanisms, which is represented by the different shape of the curves. The steeper slope of CH_LA curves at lower concentrations indicates stronger antibacterial activity in the whole pH range (Figure 4b).

In the case of *E. coli*, at the highest concentration at all media pH levels, as well as at the whole concentration range at pH 4 and 4.5, CH_LA exhibits stronger antibacterial activity (Figure 5, Tables S2 and S3). At pH 5 and above, the activity of CH and CH_LA is comparable. The shape of the curves is similar (practically at all concentrations, for pH 5 to pH 6) which implies that both materials interact with bacteria in a similar manner, despite the molecular structure differences, which is opposite to the above-presented Gram-positive *S. aureus* results.

The differences in the antibacterial abilities of CH and CH_LA are much more pronounced in the case of *H. pylori*. Both, the shape of the curves as well as the values of living cells clearly point that CH_LA has higher activity against bacteria at pH 5 and 5.5 (Figure 6, Table S4).

Summarizing, both CH and CH_LA significantly inhibited the growth of *E. coli* and *S. aureus* in all analyzed concentrations at pH 5. In the case of *H. pylori*, the % of living cells was significantly lower compared to control in all analyzed concentrations of CH_LA and in the concentration range from 20 mg/mL to 0.312 for CH (Table S5, Figure S1).

### 3.4. Dynamic Light Scattering

To simulate hydrodynamic behavior of macromolecules in bacteria cultured media at different pH Z-average hydrodynamic radius of CH and CH_LA in solutions was measured (Table 3). The presented values are mainly for illustrative purposes since dissolving processes of the CH and CH_LA was limited due to the adverse pH values.

High values of the Z-average hydrodynamic radius observed for CH are a result of the strong tendency of chitosan molecules to aggregation at higher concentration and at increased pH values. From the literature such behavior is related with the hydrophobic interactions of *N*-acetyl-*D*-glucosamine units and hydrogen bonds between *D*-glucosamine units, observed even at very low concentration such as 1.2 × 10^−3^ base-mol/L [38]. For the CH_LA solutions the observed Z-average hydrodynamic radii were significantly higher than for CH.

## 4. Discussion

Regardless of the mechanism of chitosan-bacteria interactions direct contact of them is required. Therefore, the microdilution test was proposed, which allowed to verify the antimicrobial activity of CH and CH_LA, despite their low diffusion rate. Besides that, the tests were conducted in the range of 4–7 pH since it influences the macromolecules behavior and corresponds to various natural environments. For example, *H. pylori* can survive at stomach pH (even at 1.2) due to its adaptation abilities, that is, creating a specific microenvironment around the bacteria colonies in which the pH is nearly neutral. It is a result of production and secretion of large amounts of the urease enzyme, which catalyzes urea hydrolysis to NH_3_ and CO_2_. Released gases locally increase the pH of the bacteria environment up to neutral value. High resistance to low pH of *E. coli* and *H. pylori* allows passage through the stomach at low infective dose (as few as 10 cells), which makes those strains highly pathogenic [37]. Presented results showed that depending on the pH level, different CH or CH_LA interactions with bacteria were observed. The highest activity of CH and CH_LA against *S. aureus* compared to activity against *E. coli* and *H. pylori* might be in contradiction to the majority of literature data [39,40]. However, there is no unambiguous direction of chitosan activity against Gram-positive or Gram-negative bacteria. Some authors also reported predominant activity towards Gram-positive bacteria [41,42]. Similar level of antibacterial action of CH and CH_LA suggests that grafting of fatty acid has no significant influence on this feature.

In the case of *E. coli*, at the highest concentration at all media pH as well as at the whole concentration range at pH 4 and 4.5, CH_LA exhibits stronger antibacterial activity. This indicates that the presence of fatty acids or changed macromolecular behaviour in solution can play an important role in the interaction with the Gram-negative bacteria. It was also demonstrated in the test with *H.pylori* strain in which CH_LA exhibited stronger antibacterial properties. This result is especially interesting in light of the fact that, in the literature, chitosan action against Gram-negative bacteria is mainly related with the amino groups presence. Nevertheless, the amount of amino groups in *N*,*O*-acylated derivative—CH_LA is lower than in CH. Therefore, we assume that the specific chitosan/chitosan derivatives various macromolecules arrangement should also be taken into consideration in all antimicrobial investigations. Chitosan with different deacetylation degrees (thus, different amounts of free amino groups) will have changed special conformation, arising from changed inter- and intramolecular interactions [43].

The differences in the values of hydrodynamic radius of CH and CH_LA were measured using the dynamic light scattering method. These differences were a consequence of lower solubility of CH_LA in aqueous environment and thus higher aggregation. The tendency to aggregation was responsible for the lack of noticeable antibacterial activity of chitosan and its derivative in the diffusion tests due to hindered mobility (low diffusion coefficient, Table 3) in the bacteria cultured media. Therefore, the planned application thus working conditions should be taken into account for critical evaluation of antibacterial properties of chitosan derivatives. According to the DLS results, although the CH_LA is not able to diffuse into the agar environment and to show positive results in the diffusion tests (no inhibition zone), strong and broad antibacterial activity has been confirmed in the microdilution test where the liquid environment allows to direct contact of macromolecules and bacteria.

As it was mentioned above, grafting of long fatty acid chains (hydrophobic molecules) changed the behavior of macromolecules in water solutions including its swelling properties. Therefore, varied behavior of CH and CH_LA was observed in the mucin interactions test. It is well known that the interaction of unmodified chitosan and mucin is based mainly on electrostatic complexation. The interaction strength depends not only on the factors related to the intrinsic features of chitosan and mucin, but also on solution properties, e.g., concentration, pH or ionic strength [21]. In general CH_LA protonation of amino groups is reduced due to the consumption of some of the amino groups in the *N*-acylation reaction with the fatty acid. All of that is reflected in the difference of the absorbance measurements for CH_LA. Hypothetically, the hydrophobic-hydrophobic interactions are the driving force for CH_LA_MUC strong interactions. Mucin type II exhibits the amphiphilic nature due to the presence of hydrophilic polysaccharide side chains and hydrophobic domains in the structure, such as Von Willenbrand C- and D-domains rich in cysteine and aminoacids [22]. This amphiphilic character is responsible for the formation of the mucin gel network. The hydrophobic domains can interact with hydrophobic fatty acids of CH_LA, probably leading to an interpenetrating gel network formation.

## 5. Conclusions

In this work, *N*,*O*-acylated chitosan derivative was tested in terms of mucoadhesiveness and antibacterial activity towards *S. aureus*, *E. coli* and *H. pylori* bacteria strains. The CH_LA—mucin interactions depended strongly on components weight ratio, where the hydrophobic-hydrophobic interactions seemed to be the driving force for this interactions. In contrast, CH—mucin system stability was mainly due to electrostatic complexation. The antibacterial activity of CH and CH_LA was influenced by polymer concentration and media pH. The results showed that CH_LA exhibited stronger antibacterial properties against all the tested microorganisms compared to CH. It was confirmed that Gram-positive bacteria *S. aureus* was more sensitive to CH and CH_LA than Gram-negative bacteria *E. coli* and *H. pylori*. At pH 5 and the highest concentration, the average % of living bacteria cells was 8% for *S. aureus*, 22% for *E. coli* and 32% for *H. pylori* for CH_LA material. The mucoadhesive behavior and enhanced antibacterial activity of CH_LA was a result of amphiphilic character and changed hydrodynamic behavior as confirmed by DLS analysis.

## Figures and Tables

**Table 1:** Antibacterial properties of CH and CH_LA determined by agar impregnation method.

		CFU × 10^6^/mL		
	Concertation [mg/mL]	*E. coli*	*S. aureus*	*H. pylori*
CH	10	70 ± 2	42 ± 2	61 ± 3
	5	67 ± 3	39 ± 3	60 ± 3
	2	69 ± 4	40 ± 6	59 ± 6
	1	71 ± 3	42 ± 4	66 ± 5
CH_LA	10	68 ± 3	50 ± 3	61 ± 3
	5	71 ± 6	47 ± 2	65 ± 5
	2	73 ± 5	49 ± 1	60 ± 6
	1	69 ± 4	51 ± 3	63 ± 5
	Control	70 ± 2	41 ± 2	62 ± 6

**Table 2:** Growth of *S. aureus, E. coli* and *H. pylori* in media with different pH levels.

pH of Growth Medium	Bacteria *		
	*S. aureus*	*E. coli*	*H. pyroli*
7.0	0.61 ± 0.05	2.26 ± 0.08	1.19 ± 0.08
6.5	0.70 ± 0.04	2.34 ± 0.04	1.26 ± 0.04
6.0	0.71 ± 0.07	2.46 ± 0.06	1.36 ± 0.06
5.5	0.73 ± 0.08	2.34 ± 0.04	1.15 ± 0.04
5.0	0.67 ± 0.10	2.41 ± 0.09	1.18 ± 0.06
5.5	0.03 ± 0.06	2.73 ± 0.06	0.37 ± 0.09
4.0	0.03 ± 0.07	2.22 ± 0.06	0.37 ± 0.06

* Data are presented as a mean of absorbance after incubation.

**Table 3:** Z-average hydrodynamic radius, size distribution and diffusion coefficient of CH and CH_LA in medium with different pH.

	pH	Z-Average R_h_ [nm]	PdI	Peak 1 Mean Size [nm]	Peak 1 Area [%]	Peak 2 Mean Size [nm]	Peak 2 Area [%]	Diffusion Coefficient [µm^2^/s]
CH	4	343 ± 32	0.39 ± 0.05	199 ± 10	83 ± 1.0	30 ± 3	17 ± 1	1.9 ± 0.2
	5	302 ± 118	0.40 ± 0.06	164 ± 42	87 ± 4.8	27 ± 14	13 ± 5	2.4 ± 0.8
	7	248 ± 49	0.38 ± 0.04	180 ± 33	87 ± 2.8	35 ± 11	13 ± 3	2.7 ± 0.5
CH_LA	4	4990 ± 1760	0.49 ± 0.20	3835 ± 1380	100	-	-	0.13 ± 0.05
	5	6220 ± 795	1	N/A	N/A	N/A	N/A	0.10 ± 0.02
	7	4644 ± 970	1	N/A	N/A	N/A	N/A	0.15 ± 0.03

Where: R_h_—hydrodynamic radius; PdI—polydispersity index; N/A—values not available from the software.

## Data Availability

Data is contained within the article.

## Supplementary Materials

The following are available online at https://www.mdpi.com/2073-4360/13/1/107/s1, Table S1: Antibacterial properties of CH and CH_LA depending on the pH of the culture medium against *S. aureus*, Table S2: Antibacterial properties of CH depending on the pH of the culture medium against *E. coli*, Table S3: Antibacterial properties of CH_LA depending on the pH of the culture medium against *E. coli*, Table S4: Antibacterial properties of CH and CH_LA depending on the pH of the culture medium against *H. pylori,* Table S5: Statistical differences in % of living cells treated with CH and CH_LA compared to control (non- treated cells) in pH 5, Figure S1: Antibacterial properties of CH and CH_LA at pH 5 of the culture medium against (a) *S. aureus*, (b) *E. coli*, (c) *H. pylori*.
