# Supplementary material for: Antibacterial Activity of N,O-Acylated Chitosan Derivative
Source: Polymers (Basel). 2020 Dec 29;13(1):107. doi: 10.3390/polym13010107 (PMC7794783; doi:10.3390/polym13010107)
Supplement: Supplementary file 1 [file polymers-13-00107-s001.pdf]

Supplementary Materials

Table S1. Antibacterial properties of CH and CH_LA depending on the pH of the culture medium against *S. aureus.*

Concentration [mg/mL]	CH			CH_LA		
	pH 5.0	pH 5.5	pH 6.0	pH 5.0	pH 5.5	pH 6.0
**20**	8±2.25	18±1.41	20±1.67	8±1.47	11±0.41	20±1.50
**10**	15±1.68	26±1.89	34±2.88	7±1.38	24±2.41	29±2.11
**5**	22±1.84	43±1.96	60±1.84	12±1.38	33±1.95	38±1.84
**2.5**	28±2.54	53±2.04	78±1.49	24±0.27	38±1.58	45±1.65
**1.25**	42±0.92	67±2.73	96±1.38	29±1.98	47±1.97	55±2.43
**0.625**	48±1.45	76±2.00	99±2.58	36±0.81	52±2.00	65±1.73
**0.312**	57±1.46	87±2.55	100±1.00	43±1.60	63±2.88	76±2.48
**0.156**	63±0.90	97±1.07	101±0.69	58±1.72	73±2.00	85±1.77
**0.078**	75±1.60	100±1.06	100±1.25	68±0.95	78±2.11	96±1.96
**0.039**	85±1.54	101±2.74	100±0.47	77±1.58	90±1.76	99±1.88

Data are presented as a mean of % of living cells compared to control (100%) after 48 h of incubation.

Table S2. Antibacterial properties of CH depending on the pH of the culture medium against *E. coli.*

Concentration [mg/mL]	pH 4	pH 4.5	pH 5	pH 5.5	pH 6
**20**	6±0.78	16±1.21	23±1.75	38±0.13	64±3.06
**10**	11±1.04	23±2.19	34±1.39	41±0.51	71±0.89
**5**	16±0.74	33±2.11	41±0.30	55±0.83	76±0.54
**2.5**	24±1.64	39±0.71	47±1.04	61±0.87	78±0.14
**1.25**	36±1.28	47±1.29	54±1.84	72±1.33	79±0.84
**0.625**	48±1.41	52±1.38	63±1.77	75±0.72	81±0.96
**0.312**	56±1.31	66±0.90	72±1.19	87±0.47	85±0.84
**0.156**	65±1.55	72±2.14	77±1.22	95±0.47	91±0.98
**0.078**	72±1.77	80±1.75	84±2.79	98±0.44	99±0.77
**0.039**	82±1.43	93±1.37	92±1.03	100±0.47	100±0.47

Data are presented as a mean of % of living cells compared to control (100%) after 24 h of incubation.

Table S3. Antibacterial properties of CH_LA depending on the pH of the culture medium against *E. coli.*

Concentration [mg/mL]	pH 4	pH 4.5	pH 5	pH 5.5	pH 6
**20**	3±0.96	10±1.19	22±1.48	33±1.22	55±0.93
**10**	7±1.13	16±1.30	34±1.05	38±1.09	72±1.11
**5**	17±1.14	21±1.22	43±1.01	46±0.68	74±3.18
**2.5**	27±1.27	36±0.66	49±0.64	55±2.46	77±1.72
**1.25**	32±1.32	43±2.60	56±1.05	67±0.63	84±1.55
**0.625**	40±1.37	48±1.13	66±1.21	76±1.97	91±0.60
**0.312**	47±0.79	58±1.68	69±0.81	79±0.63	92±1.28
**0.156**	59±2.60	73±1.45	79±0.35	88±0.40	95±1.15
**0.078**	66±2.26	82±1.22	83±1.35	94±1.45	100±0.82
**0.039**	77±1.61	86±4.56	94±0.58	100±0.47	100±0.47

Data are presented as a mean of % of living cells compared to control (100%) after 24 h of incubation.

Table S4 Antibacterial properties of CH and CH_LA depending on the pH of the culture medium against *H. pylori.*

Concentration	CH			CH_LA		
[mg/mL]	pH 5.0	pH 5.5	pH 6.0	pH 5.0	pH 5.5	pH 6.0
**20**	32±1.32	55±1.31	73±1.99	23±0.67	34±1.08	62±1.10
**10**	42±1.29	65±1.72	87±1.36	32±0.78	42±1.27	77±1.42
**5**	59±0.68	74±1.27	92±1.08	46±1.10	50±1.07	78±1.13
**2.5**	63±1.28	79±0.12	98±0.63	52±0.98	54±0.82	86±1.93
**1.25**	69±0.46	82±1.36	99±0.94	55±1.14	62±1.28	88±1.58
**0.625**	77±1.41	94±1.17	100±0.82	59±0.35	68±1.65	92±1.58
**0.312**	87±1.13	97±0.32	100±0.47	67±0.73	89±1.07	95±2.82
**0.156**	99±0.40	100±0.47	100±0.82	77±1.50	98±0.71	97±1.24
**0.078**	100±0.47	100±0.82	101±0.94	93±1.28	99±1.22	99±0.73
**0.039**	100±0.47	100±0.47	100±0.82	96±0.22	99±0.84	100±1.25

Data are presented as a mean of % of living cells compared to control (100%) after 24 h of incubation.

Table S5. Statistical differences in % of living cells treated with CH and CH_LA compared to control (non- treated cells) in pH 5.

Concentration (mg/L)	*S. aureus*		*E. coli*		*H. pylori*	
	CH	CH_LA	CH	CH_LA	CH	CH_LA
20	****	****	****	****	****	****
10	****	****	****	****	****	****
5	****	****	****	****	****	****
2.5	****	****	****	****	****	****
1.25	****	****	****	****	****	****
0.625	****	****	****	****	****	****
0.312	****	****	****	****	****	****
0.156	****	***	****	****	ns	****
0.078	****	**	****	****	ns	****
0.039	****	*	***	****	ns	*

ns - no statistically significant differences, **** - $p < 0.0001$, *** - $p < 0.001$, ** - $p < 0.01$, * - $p < 0.05$

a)

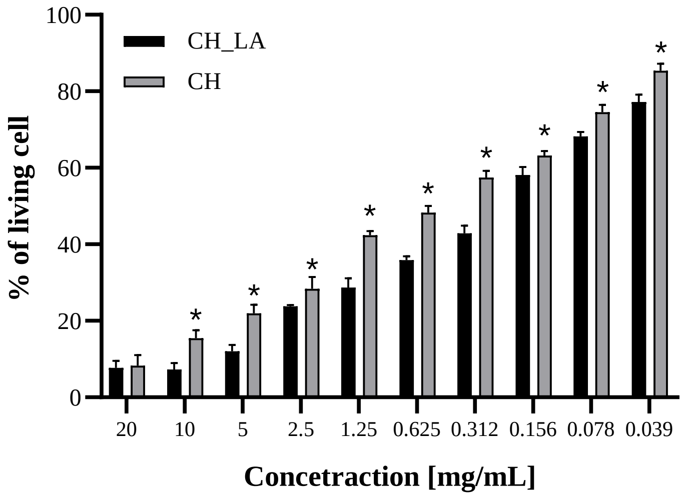

b)

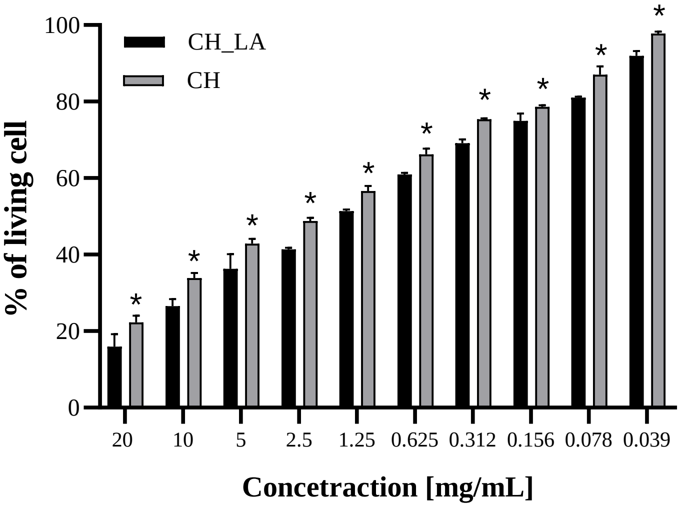

c)

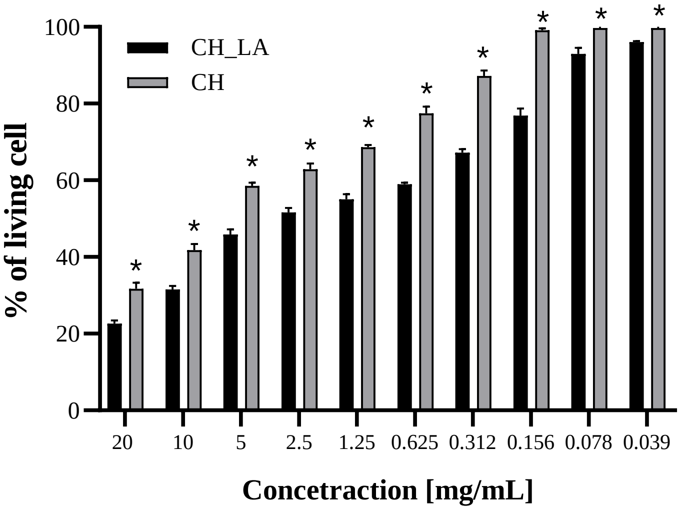

Figure S1. Antibacterial properties of CH and CH_LA at pH 5 of the culture medium against (**a**) *S. aureus,* (**b**) *E. coli,* (**c**) *H. pylori.* Data are presented as mean ± standard error of the mean (SEM); * - values significantly different $p < 0.05$.
